# Chondroprotection and Molecular Mechanism of Action of Phytonutraceuticals on Osteoarthritis

**DOI:** 10.3390/molecules26082391

**Published:** 2021-04-20

**Authors:** Stanislav Sukhikh, Svetlana Noskova, Svetlana Ivanova, Elena Ulrikh, Alexsander Izgaryshev, Olga Babich

**Affiliations:** 1 Institute of Living Systems, Immanuel Kant Baltic Federal University, A. Nevskogo Street 14, 236016 Kaliningrad, Russia; stas-asp@mail.ru (S.S.); svykrum@mail.ru (S.N.); olich.43@mail.ru (O.B.); 2 Department of Bionanotechnology, Kemerovo State University, Krasnaya Street 6, 650043 Kemerovo, Russia; a.izgarishev@mail.ru; 3 Natural Nutraceutical Biotesting Laboratory, Kemerovo State University, Krasnaya Street 6, 650043 Kemerovo, Russia; 4 Department of General Mathematics and Informatics, Kemerovo State University, Krasnaya Street 6, 650043 Kemerovo, Russia; 5 Kuzbass State Agricultural Academy, Markovtseva Street 5, 650056 Kemerovo, Russia; elen.ulrich@mail.ru

**Keywords:** osteoarthritis, phytonutraceuticals, molecular action, chondroprotection

## Abstract

Osteoarthritis (OA) is a degenerative joint disease and an important cause of incapacitation. There is a lack of drugs and effective treatments that stop or slow the OA progression. Modern pharmacological treatments, such as analgesics, have analgesic effects but do not affect the course of OA. Long-term use of these drugs can lead to serious side effects. Given the OA nature, it is likely that lifelong treatment will be required to stop or slow its progression. Therefore, there is an urgent need for disease-modifying OA treatments that are also safe for clinical use over long periods. Phytonutraceuticals are herbal products that provide a therapeutic effect, including disease prevention, which not only have favorable safety characteristics but may have an alleviating effect on the OA and its symptoms. An estimated 47% of OA patients use alternative drugs, including phytonutraceuticals. The review studies the efficacy and action mechanism of widely used phytonutraceuticals, analyzes the available experimental and clinical data on the effect of some phytonutraceuticals (phytoflavonoids, polyphenols, and bioflavonoids) on OA, and examines the known molecular effect and the possibility of their use for chondroprotection.

## 1. Introduction

Osteoarthritis (OA) is the leading cause of disability for millions of people [1,2], the treatment of which requires billions of dollars of economic investment. OA affects bones, synovium, meniscus, ligaments, tendons, and articular cartilage [3,4], which plays an essential role in the amortization of the surfaces of joints and bones. Due to the destruction of cartilage, OA leads to bone friction, pain, and, ultimately, loss of the ability to move [5]. The main goal of OA therapy in progressive degeneration of articular cartilage is to alleviate the symptoms and consequences of this complex disease [3,6,7].

Currently, there is no effective treatment for OA, only the therapy that slows or prevents OA progression [6,8]. So far most of the treatment methods (prescribing analgesics or nonsteroidal anti-inflammatory drugs (NSAIDs)) have focused on relieving pain and improving joint function without the possibility of solving the problem of the OA progression and complications [9,10]. Moreover, long-term use of drugs that alleviate the OA symptoms leads to side effects affecting the gastrointestinal tract, kidneys, and cardiovascular system [9,11]. Given the nature of OA, patients with this disease require lifelong therapy. The urgent need for the OA symptom-relieving treatment method suitable for long-term use with minimal side effects in clinical practice is, of course, obvious.

Nutraceuticals are supplements that have medical and/or health benefits when used as part of preventive and therapeutic interventions. They are considered not only as a safe alternative to existing drugs but also as drugs to relieve OA symptoms. Over-the-counter alternatives (dietary supplements and phytonutraceuticals) are used to treat 47% of OA patients [12]. Recent research has shown that plant flavonoids, polyphenols, and bioflavonoids are naturally occurring compounds found in fruits, tea, spices, wine, and vegetables. They have anti-inflammatory and antimetabolic effects on the OA symptoms and a great potential for protection against oxidative stress [13].

This review examined the clinical effects and potential mechanisms of action of nutraceuticals traditionally used in the OA treatment. We focused attention on phytonutraceuticals (plant flavonoids, polyphenols, and bioflavonoids), which strongly showed their efficacy in the OA treatment in in vitro and preclinical studies but have not been sufficiently studied in clinical trials. At the end of the review, a new targeted phytonutraceutical-based approach to effectively prevent or stabilize OA disease was discussed.

## 2. The Effectiveness and Mechanism of Action of Modern Phytonutraceuticals

For a long time, safe nutraceuticals have attracted attention due to their potential in disease therapy. Dietary macronutrients, including protein and amino acids, fatty acids (such as omega-3), vitamins, and some minerals, are not only the foundation of biological processes but also have a positive effect on the structure and function of joints [13,14,15]. The biological functions of vitamins differ significantly, which is reflected in the variety of syndromes that occur when one vitamin is deficient [16]. Lack of vitamins can also affect the development of osteoarthritis (Table 1). Various vitamin deficiencies have been identified in OA patients, including decreased vitamin C and D levels in blood serum [17,18]. One study found that subjects with recommended or higher intakes of vitamin C were 1.9 times less likely to have hip OA [19], and another study found that 24% of patients with advanced OA were vitamin D deficient [20]. Increased vitamin C intake, an antioxidant vitamin found in many fruits and vegetables, reduces cartilage loss and the risk of OA progression in patients with this condition. While vitamin D_3_ is a central regulator of mineral homeostasis [21]. Thus, understanding the effect of vitamin deficiency on joint health and the importance of nutritional supplements for OA prevention or treatment is of significant scientific and clinical interest. Avoiding “healthy foods,” as well as consuming foods high in fat and sugar, can exacerbate the disease [14]. In other words, the ingredients in nutraceuticals are essential to keep joints healthy, and some ingredients–to prevent the onset and development of osteoarthritis. Nutraceuticals such as olibanum, *Harphygophytum procumbens*, *Phytodolor*, willow bark, and such supplements as green mussels, glucosamine, chondroitin, collagen hydrolyzate, lipids (avocado/soy non-saponifiables), and essential fatty acids are also used for prevention and the treatment of OA (Table 1). Glucosamine and chondroitin sulfate are some of the most common healthy foods used to treat OA. Glucosamine is an amino sugar originally isolated from shellfish chitin and is an important part of glycosaminoglycan chains and proteoglycans (the main protein of the extracellular matrix of cartilage) [22]. Chondroitin sulfate is a glycosaminoglycan used for the synthesis of proteoglycans [23]. Many researchers have studied the efficacy of glucosamine, chondroitin sulfate, or a combination of both in the OA treatment, and research data show that these drugs improve symptoms and structural function of the joints compared to placebo [24,25]. The results of these clinical trials may be related to the complexity of OA treatment, dosage and efficacy, route of administration, and product quality. Understanding the mechanism of action of glucosamine and chondroitin sulfate can provide better guidance for clinical use [26].

## 3. Basic Phytonutraceuticals

Studies [12,28] show that rosehip berries, ginger root, green tea leaves, turmeric root, pomegranate peel, containing a significant amount of polyphenols and phytoflavonoids, have chondroprotective activity, which positively affects the OA prevention and treatment.

### 3.1. Green Tea

Green tea is a source of polyphenols, including epigallocatechin-3-gallate, and one of the most widely consumed drinks in the world [65,66]. The antioxidant activity of epigallocatechin-3-gallate is hundreds of times higher than the antioxidant activity of natural vitamins [67,68]. Further studies on the efficacy of green tea leaf and shoot phytoflavonoids in the treatment of human OA are planned, but studies in mice and rabbits have shown the need to confirm the efficacy of green tea therapies in clinical trials.

It was found that the introduction of epigallocatechin-3-gallate into the drinking water of mice with arthritis significantly mitigated the course of the disease. The observed effect was associated with a decrease in the inflammatory indicators TNF-α, COX-2, and a decrease in type I and II IgG immunoglobulins, which indicates a decrease in the inflammatory immune response. Daily consumption of green tea extracts with drinking water slowed the progression of adjuvant-induced arthritis in rats, suppressed serum IL-17 levels, and increased serum IL-10 levels [67,68]. Since cartilage destruction is a sign of OA, and inflammation plays a significant role in accompanying OA, green tea extracts may have good potential in OA prevention and treatment.

### 3.2. Pomegranate

In folk medicine, pomegranate has long been used to treat inflammatory processes in OA [69,70]. The high content of polyphenols and tannins endows pomegranate with significant antioxidant activity [65,71]. Pomegranate anthocyanins and polyphenols have an anti-inflammatory effect. It is known [12,28] that high doses of pomegranate juice injected into mice with OA reduced joint damage and promoted proteoglycan preservation. The results obtained in vivo confirm that pomegranate has a positive effect on the cartilage destruction in OA.

### 3.3. Ginger

Ginger has long been used in OA treatment in China and India. It is known that ginger reduces inflammation and stimulates blood circulation [67,72]. The US Food and Drug Administration (FDA) has recognized the safety of ginger [12,28]. The beneficial effect of using ginger as an alternative to drug therapy in arthritis treatment has been proven. Comparison of ginger and ibuprofen in placebo-controlled studies showed that joint inflammation was significantly reduced in both groups. No differences were found between the use of ginger and placebo. A placebo-controlled study on the use of ginger and galangal extracts in OA showed that the groups receiving the ginger and galangal extracts had a significant reduction in pain symptoms compared with the placebo groups.

### 3.4. Turmeric

Since ancient times, Asian countries use turmeric as a spice, safe and beneficial for the body [70,73]. About 90% of the biologically active substances in turmeric is curcumin. Currently, there are no reliable clinical results on the positive effect of curcumin on OA treatment; however, an in vitro study [12,28] proved its anti-inflammatory effect in OA. All study participants who received curcumin noted its positive effects on reducing pain and increasing joint mobility, in contrast to the placebo group [28,38].

### 3.5. Rosehip

Rosehip powder is widely used in traditional medicine [71,73]. A meta-analysis of randomized controlled trials (RCT) has shown that rosehip powder reduces pain and reduces the use of analgesics in OA patients [51]. A long-term clinical trial comparing the effects of different dosages of rosehip in patients with knee OA is currently underway [12,26].

## 4. Phytonutraceuticals for Molecular Effects on OA

### 4.1. Key Molecular Targets in OA

Several key molecules have been identified as targets for the OA treatment [72,74]. Cytokines play an important role in OA development, one of which is IL-1β. This cytokine is responsible for cartilage degradation and joint destruction. The cytokine IL-1β causes changes and destruction of the structure of cartilage protein and cells, and it suppresses the formation of hyaline cartilage collagen. It has been proven that the IL-1β content in OA patients is significantly higher and directly proportional to the OA index. The IL-1β content is a reference indicator in the OA diagnostic process. The cytokine IL-1β is an important therapeutic target in OA. Another important cytokine in OA is cathepsin B, which degrades aggrecan in matrix metalloproteinase [73,75].

It is also known that MMP plays an important role in the development of both OA and polyarthritis (PA). This study examined the levels of MMP-1, -2, -3, -7, -8, -9, and -13 in 97 PA patients and 103 OA patients. The results showed that the levels of MMP-1, MMP-2, MMP-3, MMP-8, and MMP-9 were higher in PA patients than in OA patients. Moreover, among all tested MMPs, the level of MMP-3 was very high [74,76]. The early degenerative stage of OA is usually characterized by increased levels of cathepsin B, which can destroy aggrecan in the proximity of MMP-3, which is the cause of the OA pathogenesis. The highest level was recorded for metalloproteinase-3. It was found that the increased OA pathogenesis is caused by a high level of cathepsin B, which destroys aggrecan in metalloproteinase-3. Circadian rhythm signaling, as well as the tyrosine pathway, are key pathways in OA.

When identifying the expressed genes of OA patients and healthy people, their microarray was analyzed, and it was found that platelet growth factors, β-polypeptide, and interferon-γ can be targets in OA diagnosis and treatment [75,77]. The expression of proteolytic enzymes and cartilage degradation in OA are reduced by hyaluronic acid derivatives, double integrin, and IL-6, which increase the production of IL-10 cytokines, which exhibit anti-inflammatory effects. It was shown that the secretion of IL-1β and IL-8 and inflammation are reduced by a hyaluronic acid derivative, cetylamide. It slows down the production of NF-kB [72,74,76,78].

The differentiation of cells into effector T cells or regulatory T cells, B cells, or monocytes occurs under the action of IL-6 cytomine [77,79]. T cells produce 25 cytokines, including TNF-α (TNF-α), IFN-γ, and IL-17A cytokines. A subpopulation of T-helper cells limits the production of IL-17A. The receptors for the transcription factor CRAR and the chemokine CCR6 restrict IL-17A to memory T cells [78,80]. In RA patients, CCR6 + Th cells are found during inflammation of the synovial membrane, and the proportion of CCR6 + in peripheral blood increases; Th cells are found in patients with early OA. Studies have also shown that pathogenic signatures are not limited to cells that produce high levels of IL-17A, and even populations of CCR6 + Th cells that produce small amounts of IL-17A have pathogenic signatures and activities [79,81]. Therefore, the IL-17 producing cell population may be an important target for OA treatment.

IL-17, a low molecular weight transmembrane glycoprotein, plays an important role in OA inflammation [80,82]. Further inflammation is facilitated by the transduction and expression of the cytokines IL-1, IL-6, and IL-8. The expression of IL-17 in OA patients correlated with IL-15, and the latter promoted the production of IL-17 in the joints by mononuclear cells [81,83]. It has been shown that, upon stimulation of the secretion of IL-6 and IL-8, TNF-α and IL-17 cells exhibit combined effects, stimulate granulocytes that cause joint inflammation and cartilage degradation. A therapy that targets individual cytokines is not very effective because many molecules play an important role in OA development, leading to inflammation and destruction of bones and cartilage. Recent studies of anti-TNF-α/IL-17 bispecific antibodies show that inhibition of TNF-α and IL-17 is more effective than monotherapy in suppressing cytokines, chemokines, and MMPs and blocking tissue destruction in OA [82,84].

Other studied cytokines also play an important role in joint inflammation. Studies have shown that joint health of collagen-induced arthritis (CIA) mice lacking urokinase-type plasminogen (uPA) is almost completely improved, which is mainly associated with significant local mRNA levels of key inflammatory mediators (such as TNF-α). In inflammatory diseases of the joints, proteolysis associated with the cell and mediated by the uPA receptor signaling the bone marrow cells is important. In OA treatment, key proteolysis and signal transduction can become an innovation in strategies to prevent cartilage degradation and joint inflammation [83,85]. Oncostatin M (OSM) is another cytokine that has become an effective OA mediator. IL-1 is known to slow down the damage to articular cartilage in mice and has anti-inflammatory properties. OSM is an effective IL-6 inducer and increases the activity of uPA and P-selectin in synovial fibroblasts. Based on the data obtained in studies, OSM is a promising target for OA therapy [84,86].

Proteins that play an important role in OA treatment include type I, II, III collagens, MMPs, and aggrecan [85,87]. Proteins that play an important role in OA treatment include type I, II, III collagens, MMPs, and aggrecan [85,86]. The differentiation of bone marrow osteoblasts is facilitated by type I collagen, which reacts with α2β1 integrin and transmits an intercellular signal. The expression of osteoblasts is inhibited by antibodies α2, and integrin α2β1 is inhibited by type I collagen and peptides [87,88]. The cartilage integrity is maintained by type II collagen when it forms a fibrous network damaged by OA [89,90]. Collagen produced by chondrocytes has been reported to be associated with the degradation and denaturation of type II collagen, resulting in an OA increase [87,90]. Aggrecan is also important for the regulation of cartilage destruction. The proteases aggrecanase-1 and aggrecanase-2 destroy aggrecan. Plasmid vectors of shRNA expression for aggrecanases were transfected into rat chondrocytes. The results showed that the inhibitory effect of RNA interference on aggrecanase-1 and aggrecanase-2 can reduce aggrecan degradation without affecting normal cell functions [88]. Collagen produced by chondrocytes has been reported to be associated with the degradation and denaturation of type II collagen, increasing OA [87,90]. Aggrecan is also important for the regulation of cartilage destruction. The proteases aggrecanase-1 and aggrecanase-2 destroy aggrecan. Plasmid vectors of shRNA expression for aggrecanases were transfected into rat chondrocytes. The results showed that the inhibitory effect of RNA interference on aggrecanase-1 and aggrecanase-2 can reduce aggrecan degradation without affecting normal cell functions [88,89]. In a study [87,90], it was reported that aggrecanase inhibition leads to the proliferation of chondrocytes, an increase in the amount of aggrecan, and an increase in the amount of type II collagen through RNA interference. Therefore, it can be concluded that these proteins can be used to control OA progression. Studies [90,91,92,93] showed that bone marrow stromal cells and fibroblast growth factors stabilize the amount of aggrecan under the action of glycosaminoglycan nanoparticles.

### 4.2. Anti-Inflammatory Effects of Phytonutraceuticals

Pomegranate extracts have an anti-inflammatory effect, inhibiting the activity of NF-κB, COX-2, and PGE 2 [93,94]. In this case, the active components are prodelphinidins-condensed tannins. Gallocatechin dimers and trimers and gallocatechin-epigallocatechin significantly reduce PGE2 synthesis. The inhibition of PGE2 synthesis was confirmed by an in vitro test on purified COX enzymes, and the selectivity of prodelphinidins towards COX-2 was found [95]. Pomegranate extract blocks IL-1β-induced activation and DNA-binding activity of NF-κB, inhibiting phosphorylation of its inhibitor IκB-α in human OA chondrocytes [94,96].

Brazilin, which is a part of *Caesalpinia Sappan L.* extract (CSE), suppresses the expression of inflammatory mediators IL-1β, iNOS, COX-2 and TNF-α, NO, and PGE 2 [30,31]. The active component of *Harpogophytum procumbens*, harpagoside, inhibits the release of IL-1β, IL-6, TNF-α, and PGE [40].

Prodelphinidin, a condensed polymeric tannin found in pomegranate, inhibits the synthesis of PGE 2 by suppressing COX-2 in human chondrocytes [97,98]. It was demonstrated that ginger extract reduces induced IL-1β and NO and PGE 2 production in OA cartilage. It was shown in [99] that ginger aqueous extract was effective in suppressing the production of TNF-α, PGE 2, and COX-2 expression in human synoviocytes by regulating the activation of NF-κB and the degradation of its inhibitor IkB-α. A decrease in IL-1β-induced TNF-α expression and TNF-α-induced COX-2 production in synoviocytes has also been reported [100]. Ginger extracts contain gingerols and dia-3-heptanoids. These compounds have been shown to act as inhibitors of COX and 5-1-lipoxygenase [101]. These enzyme systems are critical in the production of PG and leukotrienes, which are key mediators of inflammation [100].

Berries and nuts contain a polyphenolic phytoalexin—resveratrol, which suppresses NF-κB-dependent inflammation, apoptosis, IL-1β induction, inhibition of caspase-3, and suppression of the NF-κB pathway in chondrocytes [102,103,104].

It is known that the production of inflammatory mediators is suppressed by the bioactive polyphenol of green tea—epigallocatechin-3-gallate; it also suppresses PGE 2, COX-2, and NF-κB [105]. In OA, epigallocatechin-3-gallate inhibited IL6, IL-8, and TNF-α in chondrocytes and inhibited the NF-κB bond in humans [106].

Curcumin reduced the expression of NF-κB of the COX-2 gene, decreased induction of cytomine IL-1β, activation and translocation of NF-κB [12,27,69], and prevented the production of NO, PGE 2, IL-6, and IL-8 [77,107]. Rosehip preparations have anti-inflammatory properties and were shown to inhibit the expression of iNOS and IL-1α, as well as IL-1β-induced IL-1α and IL-8 in chondrocytes. The combination of glucosamine and chondroitin sulfate suppresses the expression of the COX-2 and NF-κB genes induced by IL-1 in cartilaginous explants, which leads to a decrease in the production of NO and PGE 2 [108]. One of the mechanisms by which glucosamine or chondroitin sulfate exerts an anti-inflammatory effect is the inhibition of the IL-1β-induced NF-κB pathway, which leads to a decrease in the synthesis of COX-2 [12,109].

Piperlongumine (PLM) contained in the *Piper longum* pods effectively modifies the viability of cells inhibited by IL-1β in a dose-dependent manner [110]. PLM significantly suppresses the NO and PGE2 production, iNOS and COX-2 expression, as well as the MMP-3 and MMP-13 production, and the NF-κB activation stimulated by IL-1β in human OA chondrocytes. Moreover, piperlongumine attenuates the inflammatory responses in human OA chondrocytes stimulated by IL-1β, possibly through the NF-κB signaling pathway and, for this reason, can serve as a potential anti-inflammatory agent in the OA treatment [110].

Fisetin, a natural flavonol, inhibits the IL-1β-induced inflammatory response, including the expression of NO, PGE2, TNF-α, IL-6, iNOs, COX-2, MMP-3, MMP-13, and ADAMTS-5, and reduces Sox-9 degradation, aggrecania collagen II in human OA chondrocytes [111].

### 4.3. Effects of Phytonutraceuticals on Antioxidant Stress

High levels of free oxygen radicals are produced by the inflammatory cytokines IL-1β and TNF-α [112,113]. Important signs of the OA pathogenesis are reactive oxygen species that activate pro-inflammatory cytokine genes. Epigallocatechin-3-gallate protects chondrocytes from oxidative stress and cytotoxicity [97,114], which can inhibit the activation of inflammation [106,115]. The innate antioxidant defense, the expression of catalase, superoxide dismutase, and glutathione peroxidase is increased by epigallocatechin-3-gallate [106,115]. Phytonutraceutical ginger also has a high antioxidant effect [95,106]. Gingerol, a ginger polyphenol, inhibits the release of NO and other active forms of nitrogen in macrophages [4]. The pomegranate component, anthocyanin, enhances the activity of the antioxidants catalase, superoxide dismutase, glutathione peroxidase, and glutathione reductase and is a potent antioxidant that reduces lipid oxidation.

### 4.4. Effects of Phytonutraceuticals on Anticatabolic Processes

ADAMTS is the primary OA prevention and treatment method since the overactive catabolic activity of MMPs causes cartilage degradation. Epigallocatechin-3-gallate inhibits expression of ADAMTS-1, -4 and -5 in chondrocytes, the activity of proteolytic enzymes in joint tissues, including synoviocytes and tendons, TNF-α-induced production of MMP-1 and MMP-3, IL-1β-induced expression of MMP-1, -3, and -13 in human tendon fibroblasts [114,115,116,117]. In vitro studies have confirmed an increase in the anticatabolic potential and activity of tissue inhibitors of MMP (TIMP) -1 and -2 under the action of epigallocatechin-3-gallate [95,118]. Curcumin inhibits the release of proteoglycans IL-1β, the production of MMP-1, MMP-9, and MMP-13 in tenocytes [12,27]. It was shown that curcumin reduces the MMP-3/TIMP-1 ratio by inhibiting MMP-3, does not affect the synthesis of Agg, inhibits the degradation of proteoglycans, and counteracts the stimulating effect of IL-1β on the synthesis of PGE2, NO, IL-6., IL-8, and MMP-3 [107].

Quercetin, a phenolic compound, increases TIMP-1 expression but attenuates MMP-13 expression in serum, synovial fluid, and synovial tissues. Quercetin can activate TIMP-1, suppress MMP-13, and improve OA degeneration by weakening the response to oxidative stress and inhibiting the degradation of the cartilage extracellular matrix [119].

### 4.5. Spermidine Role in OA Inhibition

The natural polyamine spermidine has a cardioprotective effect, dietary spermidine can reduce TNF-α in plasma [117] and further increase the bioavailability of NO by reducing oxidative stress in cardiomyocytes [120]. In another study, intraperitoneal injection (IP) of a spermidine analog, spermine, decreased TNF-α in the plasma of septic mice by inhibiting high motility group protein-1 and the release of inflammatory cytokines in macrophages [121]. The studies [122] focused on the role of spermidine in TNF-α-induced inflammation in OA. It was shown that spermidine significantly increased the expression of aggrecan and collagen II and decreased the expression of MMP13 in mice treated with spermidine.

It was found that spermidine treatment can significantly reduce the levels of TNF-α, IL-6, and IL-8 in the supernatant of cultured primary OA-FLS of mice [123,124]. This study proves that intraperitoneal spermidine administration can effectively reduce TNF-α expression in FLS and serum in a mouse OA model and inhibit RIP1 deubiquitination in TNF-α-mediated NF-κB/p65 activation by protein activation. All this resulted in significant suppression of synovial inflammation, while cartilage degeneration and osteophyte formation were significantly reduced, indicating the ability of spermidine to reduce the TNF-α-mediated NF-κB/p65 signaling pathway in the FLS.

### 4.6. Inhibition of Cytokines and MMP in OA

Certain proteinases such as MMP, ADAMTS (thrombospondin-motivated disintegrin and metalloproteinase), neutrophil elastase, and cathepsins (G and B) can directly damage cartilage. Depletion of proteoglycans from articular cartilage is the initial event in RA development, leading to the degradation of collagen fibrils. Interestingly, passively transferred CII-reactive monoclonal antibodies depleted a significant amount of proteoglycans within 72 h [125]. Many MMPs (MMP 1-3, MMP 7-9, MMP-13, and MT1-MMP) preferentially cleave the bond between Asn 341 -Phe 342 of aggrecan. And vice versa, ADAMTS1, ADAMTS4, and ADAMTS5 cleave Glu 373 and Ala 374 bind in addition to other sites in the G2-G3 domains of proteoglycans. Thus, the enzymes MMP and ADAMTS contribute to the degradation of aggrecan during the development of arthritis. TIMP are endogenous MMP blockers and regulators of matrix renewal, tissue reorganization, and cellular activity. Sources, targets, receptors/ligands, and main functions of MMP, ADAMTS, and TIMP involved in the pathogenesis of arthritis are summarized in Table 2 [126].

RANKL (also called TNFSF11, OPGL, TRANCE, and ODF) and its RANK receptor are essential regulators of bone repair and remodeling. Several hormones and cytokines induce RANKL production in osteoblasts and synovial fibroblasts. After binding to RANK, RANKL triggers a set of adapter molecules TRAF-6, which leads to the activation of signaling molecules such as NF-κ B, N-terminal kinase c-Jun (JNK), AKT/PKB, ERK, Src, and p38. mitogen-activated protein (MAP) kinases and transcription factor, as well as nuclear factor of activated T cells, calcineurin-dependent 1 (NFATc1) [127]. Therefore, the RANKL/RANK signaling pathway is a potential therapeutic target in osteolytic diseases. Denosumab (RANKL-specific human monoclonal antibody) is currently used to treat osteoporosis, osteosarcoma, multiple myeloma, and bone metastases [128]. Although denosumab is very specific for RANKL and good for bones, safety concerns still exist. On the other hand, the RANKL/RANK pathway also has important functions in osteoblasts. Vesicular RANK, a secretion product of mature osteoclasts, by binding to RANKL derived from osteoblasts, promotes bone formation by initiating RANKL signaling reverse, leading to activation of Runt-associated transcription factor 2 (Runx2) [129].

Initiation of the RANKL/RANK pathway induces the activation of NF-κB, which promotes osteoclast differentiation. After NF-κB stimulation, several TNF-receptor (TNFR-) factors are associated with the cytoplasmic domain-associated RANK. Among them, TRAF-6 is a prerequisite for osteoclast formation and activation [130], while the NF-κB p50 and P52 subunits modulate RANKL and TNF- and α-induced differentiation of osteoclast precursors.

The interaction between many factors generates aberrations in the recognition and activation of the immune system, causing the initiation of molecular pathways that target cartilage and bone. In this process, various immune and non-immune cells are crucial. Resident and infiltrating cells in the joints proliferate and secrete pro-inflammatory cytokines, chemokines, and matrix lysing enzymes that can destroy the joints, resulting in functional loss. Moreover, during the activation and differentiation of osteoclasts, various signaling cascades are activated that are involved in bone resorption activity. Therefore, targeting a single effector molecule is insufficient to block cartilage and bone damage in arthritis. Since OA is an immune-mediated disease, therapeutics that restore the immune balance can certainly improve clinical therapy [126].

### 4.7. The Effect of Senolytics on the Process of Cellular Aging

Senolytic drugs are agents that selectively induce apoptosis in senescent cells. These cells accumulate in many tissues with age and in places of pathology in multiple chronic diseases. In animal studies, targeting senescent cells using genetic or pharmacological approaches delays, prevents, or alleviates multiple age-related phenotypes, chronic diseases, geriatric syndromes, and loss of physiological resistance. Chronic conditions successfully treated by depletion of senescent cells in preclinical studies include weakness, cardiac dysfunction, vascular hyporeactivity and calcification, diabetes, hepatic steatosis, osteoporosis, degeneration of the spinal disc, pulmonary fibrosis, and radiation-induced damage. Senolytic agents are in the testing phase as part of a proof-of-concept clinical trial. To this end, new paradigms of clinical trials are being developed to test senolytics and other agents targeting the fundamental mechanisms of aging since the use of long-term endpoints, such as life expectancy or health maintenance, does not make sense. These strategies include testing for multiple diseases, conditions similar to accelerated aging, diseases with local accumulation of senescent cells, potentially fatal diseases associated with the accumulation of senescent cells, age-related loss of physiological tolerance, and bone fragility. If senolytics or other interventions aimed at fundamental aging processes prove to be effective and safe in clinical trials, they can transform geriatric medicine by preventing or treating multiple diseases and functional disorders in parallel, rather than one at a time [131].

Cellular senescence is usually defined as the irreversible arrest of the cell cycle and the loss of the replicative capacity of virtually all cell types that can affect tissues and possibly play an important role in the development of age-related chronic diseases and cancer. Recently, the use of naturally occurring bioactives such as resveratrol to modify the cellular aging process in tissue cells, depending on the specific context, has opened up an interesting therapeutic perspective in aging and chronic diseases such as cancer. This naturally occurring polyphenol is currently being evaluated as a promising anti-cancer and anti-aging agent. Resveratrol modulates cell cycles and multiple pathways involved in cell growth, apoptosis, aging, and inflammation, which are mainly observed in laboratory models. In vitro studies indicate that the biological effects of resveratrol on cellular aging or other cellular processes may vary depending on cell types and specific conditions [132].

Quercetin is another important senolytic. Quercetin, a member of the flavonoid family, targets PI3K/AKT и p53/p21/serpines, senescent cell anti-apoptotic pathway (SCAP) [133]. Quercetin has been shown to reduce cellular aging in vivo in epithelial and possibly other cells [134]. This may lead to an improvement in mitochondrial dysfunction (associated with aging) [135] or a decrease in senescence-associated secretory phenotype (SASP), and will lead to reducing of the pro-inflammatory pro-fibrotic microenvironment [136]. Quercetin reduces the expression of Tnf-α, IL-1 α, and MCP-1 [134]. The effects of quercetin include both direct senolysis (elimination of senescent cells) and prevention of the induction of senescence [137], since it can indirectly prevent further propagation of senescence by removing harmful senescent cells that produce SASP. Quercetin is a senolytic for radiation-induced senescent human umbilical vein endothelial cells (HUVECs) [138]. Quercetin induces aging and promotes apoptosis in a variety of cancer lines [139], and is active in delaying senescence in primary cells and rejuvenating in senescent cells. Malavolta et al. [139] suggested that the rejuvenating effect of quercetin on senescent and primary senescent cells could be pure results of senolytic activity of senescent cells and selective survival of a subpopulation of senescent cells in culture.

However, [140] reported that quercetin is unsafe for non-aging vascular endothelial cells in adults at concentrations previously reported to be safe for proliferating HUVECs. Quercetin 3-D-galactoside, an inactive quercetin derivative that needs to be cleaved by beta-galactosidase overexpressed in senescent cells, is potentially a safer senolytic [140]. Hwang et al. found that quercetin causes cell death in non-aging human coronary artery endothelial cells (HCAEC), and quercetin 3-D-galactoside is not cytotoxic to either young or senescent cells. Thus, in primary endothelial cells of an adult, quercetin and quercetin 3-D-galactoside are not senolytics.

The quercetin-related flavonoid fisetin selectively induces apoptosis in senescent but non-proliferating HUVECs. However, it is not senolytic for senescent cells of the normal human lung fibroblast cell line (IMR90) or primary human preadipocytes [141].

Piperlongumine is also a senolytic agent with the ability to selectively kill senescent cells [142]. Piperlongumine has the advantage of low toxicity and oral bioavailability [143]. One of the important molecular targets of piperlongumine is the antioxidant protein OXR1, which regulates the expression of various antioxidant enzymes. OXR1 is activated in senescent human WI38 fibroblasts. Piperlongumine binds to OXR1 directly and induces its degradation through the ubiquitin-proteasome system in a manner specific for senescent cells [144].

### 4.8. Molecular Mechanisms of Nutraceuticals Modulating Pro-Inflammatory Pathways

A unique feature of bioactive food ingredients (phytonutraceuticals) is their broad antioxidant function. Antioxidants, which have a wide range of chemical structures and activities in addition to the main food, demonstrate a variety of health benefits in preventing chronic diseases. Phytonutraceuticals are able to enhance the natural antioxidant defense system by absorbing reactive oxygen and nitrogen species, protecting and repairing DNA damage, and modulating signaling pathways and gene expression. The main pathways that are influenced by bioactive food ingredients include pro-inflammatory pathways regulated by nuclear factor kappa B (NF-κB), as well as pathways associated with cytokines and chemokines. These studies [145] summarize the importance of phytonutraceuticals and their role in the regulation of inflammatory pathways. Bioactive substances affect several physiological processes such as gene expression, regulation of the cell cycle, cell proliferation, cell migration, etc., leading to cancer prevention. The occurrence of cancer is associated with changes in metabolic pathways such as glucose metabolism, and the effect of bioactive substances in normalizing this process was provided. The onset and progression of inflammatory bowel disease (IBD), which increases the likelihood of developing colorectal cancer, can be suppressed by plant biologically active substances. Some aspects of the potential role of microRNA and epigenetic modifications in cancer development were also presented [146].

## 5. Conclusions

Phytonutraceuticals effectively suppress over-activated inflammation and catabolic activity, as well as harmful reactions caused by oxidative stress. Reduction of inflammation and antioxidant activity are essential properties of the OA treatment drugs.

It is known that phytonutraceutical relieve pain and improve joint function, thereby having a positive effect on OA symptoms, but further research on the effect of phytonutraceuticals on OA is needed. Based on the effectiveness and action of these phytonutraceutical compounds, the effectiveness of a single compound for the treatment of complex and chronic diseases with multiple risk factors such as OA may be limited. Future phytonutraceutical approaches may require a combination of compounds, and the selected compounds should have active effects on OA targets (such as inflammation and catabolism), suppress oxidative stress and relieve chronic pain, and have additional or synergistic antiarthritic effects when combined with other compounds. These new phytonutraceutical-based formulations that aim for many molecular OA targets could serve as a therapeutic strategy for a new generation of nutraceuticals in the prevention and treatment of OA.

## Figures and Tables

**Table 1:** Overview of the clinical efficacy and mechanism of action of nutraceuticals [26].

Nutraceuticals	Active Components	Clinical Efficacy	Mechanisms of Action
*Boswellia serrata*	–	Joint pain was relieved, joint swelling and stiffness decreased, joint flexion was more comfortable, and walking distance increased [27,28]	Inhibits TNF-α-induced MMP-3 expression and protects against IL-1β-induced chondrocyte death [27]
Pineapple extract	Bromelain	It did not significantly relieve pain or improve quality of life	Decreases PGE 2 expression [29]
*Caesalpinia Sappan* extract (CSE)	Brazilin (6aS, 11bR) -7,11b-dihydro-6H-indeno [2,1-c] chromene-3,6a, 9,10-tetrol)	–	Suppresses the expression of inflammatory mediators IL-1β, iNOS, COX-2 and TNF-α, NO and PGE 2; Heme oxygenase-1 mediates these effects in primary human chondrocytes stimulated by IL-1β, as well as in SW1353 cells in vitro [30,31]. CSE also suppresses the expression of MMP-1, MMP-3, MMP-7, MMP-9, and MMP-13 [32]. Подaвляeт экcпpeccию мeдиaтоpов воcпaлeния IL-1β, iNOS, COX-2 и TNF-α, CSE тaкжe подaвляeт экcпpeccию гeнов MMP-1, MMP-3, MMP-7, MMP-9 и MMP-13 [23,24]
*Caesalpinia Sappan* extract (CSE)	Sappanchalcone	–	Suppressed the production of both NO and PGE2 and suppressed the mRNA expression of TNF-α, IL-6, COX-2, and iNOS [33]
	Protosaponin D		
	Protosaponin E		
Capsaicin	–	Reducing pain and stiffness, improving joint function [34,35]	Transient receptor potential agonist vanilloid 1 (pain receptor); Long-term exposure to capsaicin leads to desensitization of pain syndrome
Cat’s claw	Quinic acid glycosides, sterols, and oxidol	Reducing pain associated with osteoarthritis [36]	Inhibits lipopolysaccharide (LPS) -induced PGE 2 production and TNF-α activation [36]
Chicory root	–	Reducing pain and reducing joint stiffness	Suppresses the production of COX-2, iNOS, TNF-α, and NF-κB [37]
Garlic extract	*Diallyl sulphide*	–	Suppresses IL-1β-induced expression of MMP-1, -3, and -13. Improves OA course in the mode of transformation of the anterior cruciate ligament of a rabbit and decreases MMP-1, –3, –13 [38]; Suppresses IL-1β-induced COX-2 expression.
Duhuo Jisheng Tang	–	Reducing pain and stiffness and improving physical function in patients with OA [39]	–
Devil’s claw	*Harpogophytum procumbens*	Relieves pain in OA patients	Suppression of TNF-α, IL-1β, IL-6 and PGE release [40]
*Phyllanthus emblica* extract	–	Relieves pain in OA patients	Suppresses the activity of hyaluronidase and collagenase type 2 in vitro and reduces GAG release in cartilage explants from OA patients. Effectively reduces the levels of pro-inflammatory cytokines, tumor necrosis factor α (TNF-α) and interleukin-1 (IL-1β) and significantly increases the concentration of anti-inflammatory cytokines (IL-10) [41]
Willow bark	Polyphenols, flavonoids, proanthocyanidins, salicin and its derivatives	Reduced OA associated pain [42,43]	Inhibits lipoxygenase (LOX-5), modulates the corresponding pro- and anti-inflammatory cytokines (interleukin 1, 6, 8, 10) and nuclear factors (TNF-α, NF-κB) [44]
**Supplements**			
Aloe vera	–	Protects the gastrointestinal tract from NSAIDs	–
Avocado/soy unsaponifiables	–	Reduced pain in OA patients and reduced consumption of NSAIDs [45,46]	Reduces levels of iNOS and MMP-13 [47]. Suppresses TNF-α, IL-1β, COX-2, and iNOS in LPS-activated chondrocytes [48]
	Calcium fructoborate	–	Suppresses IL-1β, IL-6, iNOS in vivo
	Collagen hydrolysates	Relieves pain associated with OA [49]	Stimulates the regeneration of collagen type 2 and increases the biosynthesis of proteoglycans
Edible bird’s nest extract	–	–	Reduces the expression of genes MMP-1, MMP-3, IL-1, IL-6, IL-8, COX-2, PGE2, and iNOS and increases the level of type 2 collagen, aggrecan, and SOX-9 [50]
Green-Lipped Mussel Extract	–	Reducing pain and improving knee mobility [51]	Suppresses the synthesis of the pro-inflammatory molecule leukotriene B4 and the production of PGE2 [52]
	*Lactobacillus casei*	–	Decreases TNF-α, IL-6, NF-κB, COX-2, MMP-1, −3, −13 and increases IL-4 and IL-10
	Methylsulfonylmethane (MSM)	Reducing pain symptoms and improving physical condition	Removes hydroxyl free radicals; eliminates the lack of sulfur in food, enhancing the formation of cartilage
	Polyunsaturated fatty acids (PUFA)	High N-3 PUFA levels are associated with less cartilage loss	N-3 PUFA eliminates the expression of TNF-α, IL-1β, COX-2, MMP-3, -13, ADAMTS5 in vitro [53] and protects against cartilage degradation in animals with a predisposition to OA [54]
	S-adenosylmethionine	Decreased OA associated pain intensity compared to baseline [55]	Increases proteoglycan synthesis and chondrocyte proliferation [56]
**Vitamins**			
	Niacinamide (B vitamins)	Improved joint mobility. Reducing knee pain, general pain severity, knee stiffness, and normalization of its function [50]	Niacinamide inhibits cytokine-mediated induction of NO-synthase in a number of cell types, weakens the anti-anabolic effect of IL-1 [57,58]
	Vitamin C	Improved joint mobility	Stimulates collagen and proteoglycan synthesis. Higher vitamin C intake is associated with lower mean cartilage T2, mean tibial T2, and medial tibia WORMS scores [59]
	Vitamin D	Does not affect the severity of pain or the quantitative loss of cartilage on MRI [60]; Relief of joint pain associated with OA [61]	Develops and maintains the skeleton, takes part in the metabolism of bones and cartilage [54]. Higher vitamin D intake was associated with a lower total WORMS score for cartilage and mean WORMS score for the femur [57].
	Vitamin E	Relief of pain associated with osteoarthritis and improving physical condition [61,62]	It alleviates oxidative stress in cartilaginous explants caused by mechanical stress or free radicals [62], enhances the growth of chondrocytes, and exhibits anti-inflammatory activity, plays an important role in the prevention of cartilage degeneration [63].
	Vitamin K	Relief of pain associated with osteoarthritis and improvement of physical condition	Vitamin K deficiency causes abnormal growth plate calcification and inappropriate cartilage mineralization [64]
	Vitamin A	Relief of pain associated with osteoarthritis and improvement of physical condition	Regulates the formation of cartilage and skeleton [64]

“–”: No data.

**Table 2:** MMP, ADAMTS, and TIMP in the pathogenesis of arthritis.

Enzymes/Inhibitors	Sources	Main Functions
**Metalloproteinases**		
MMP-1 *	Monocytes, fibroblasts, smooth muscle cells, chondrocytes, macrophages, endothelial cells, and keratinocytes	Releases MMP-9, promotes Akt dephosphorylation, destroys collagens I, II, II, III, VI, IX, and proteoglycans.
MMP-2	Synoviocytes, CD34^+^ vascular endothelial cells, CD68^+^ macrophages, CD14^+^ monocytes, and stromal cells	Increases VEGF expression and angiogenesis, promotes angiogenesis, and directs cartilage matrix degradation.
MMP-13 (interstitial collagenase)	Chondrocytes and macrophages	Degrades collagen fibers of types I, II, III, V, and XI, as well as basement membrane proteoglycans.
MMP-14	Macrophages, myeloid cells, FLS, and CD68^+^ osteoclasts	Facilitates the penetration of FLS into the cartilage. MT1-MMP destroys collagen types I, II, and III, laminin-1 and laminin-5, fibronectin, vitronectin, fibrin, and aggrecan, and also activates pro-MMP-2 and pro-MMP-13 on the cell surface.
**ADAMTS**		
ADAMTS-1	Chondrocyte, macrophage	Cleaves proteoglycan versican.
ADAMTS-4	Chondrocyte	Cleaves aggrecan.
ADAMTS-5	FLS, stromal cell	Cleaves aggrecan.
**Inhibitors**		
TIMP-1	Macrophages, connective tissue cells, chondrocytes, FLS, T cells, and monocytes	Weak inhibition of MMP-14, MMP-16, MMP-19, as well as MMP-24 and ADAM10. Suppresses the interaction of pro-MMP with pro-MMP-9, the formation of synovial blood vessels, the activation of MMP-3 and 9, and the invasion of synovial vessels in RA.
TIMP-2	Chondrocytes, FLS, T cells, and monocytes	Suppresses all MMP (prevents over-activation of MMP-9), ADAM12, and pro-MMP interactions with pro-MMP-2.
TIMP-3	FLS, chondrocytes, macrophages, and monocytes	Suppresses all MMP and ADAM10, ADAM12, ADAM17, ADAM28, and ADAM33; ADAMTS-1, ADAMTS-4, ADAMTS-5, ADAMTS-2 (weak); and pro-MMP interactions with pro-MMP-9 and pro-MMP-2.

* ADAMTS: disintegrin and metalloproteinase with thrombospondin motives; COMP: Cartilage Oligomeric Matrix Protein; GEP: granulin-epithelin precursor; TIMP: tissue inhibitor of metalloproteinases; vWFCP: von Willebrand factor cleavage protease; α 2M: α 2-macroglobulin.

## Data Availability

Not applicable.
